# *Journal of Educational Evaluation for Health Professions* received the Journal Impact Factor, 4.4 for the first time on June 28, 2023

**DOI:** 10.3352/jeehp.2023.20.21

**Published:** 2023-06-29

**Authors:** Sun Huh

**Affiliations:** Department of Parasitology and Institute of Medical Education, College of Medicine, Hallym University, Chuncheon, Korea; Hallym University, Korea

## 2022 Journal Impact Factor: 4.4

**Fig. 1.**
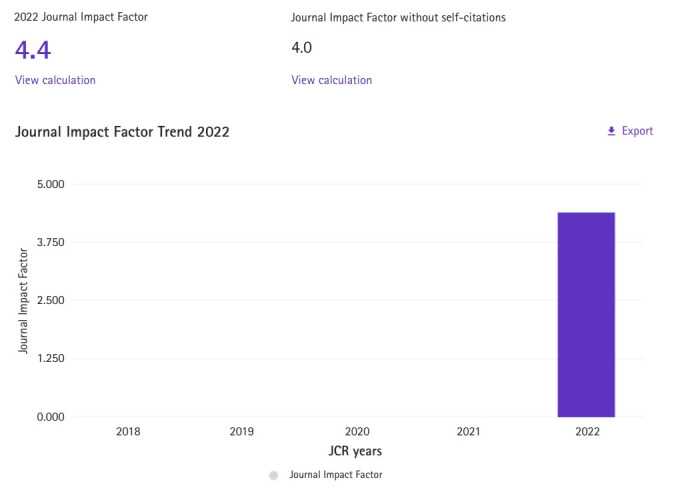
Journal Impact Factor (JIF) of the *Journal of Educational Evaluation for Health Professions* captured from the 2023 Journal Citation Report (JCR). For the Emerging Sources Citation Index journals, there are no past records available as the JIF scores are given from 2023.

As previously announced on Clarivate’s blog on July 26, 2022 [1] and March 7, 2023 [2], Emerging Source Citation Index (ESCI) journals received the Journal Impact Factor (JIF) for the first time on June 28, 2023. The 2023 Journal Citation Reports (JCR) provided JIFs for all Web of Science Core Collection journals, including ESCI and A&HCI. *Journal of Educational Evaluation for Health Professions* (JEEHP), an ESCI journal, also obtained its first JIF after 20 years of publication. I was thrilled by the impressive 2022 JIF value of 4.4 (Fig. 1). The journal ranked 7th out of 84 journals (92.9%) in the scientific disciplines education category, which is an unbelievably high value. This 2022 JIF value is comparable to the Citations per document per 2 years, 4.4 provided by 2022 Scimago Journal & Country Rank (https://www.scimagojr.com/journalsearch.php?q=19755937). This achievement was made possible through the contributions of numerous authors, reviewers, and staff from the publishing companies. Words cannot express my gratitude to them.

Naturally, a high JIF value does not guarantee the journal’s high quality; the content of the articles themselves is essential. While this simple truth is widely known, JIFs often determine a journal’s reputation in reality because many institutions or universities use JIF to measure their members’ quality of work. This type of quantitative evaluation makes it easier to demonstrate that an evaluation process is fair. In Korea, this has become a well-established trend, as persuading academics through a qualitative evaluation of each publication can be challenging.

## Which articles contributed the most to increasing the JIF?

The articles that contributed the most to the 2022 JIF are listed in Table 1. The 3 most frequently cited review articles cover topics such as statistics, the metaverse, and e-learning. The editor commissioned the articles ranked 1st, 2nd, and 5th. When I invited authors to submit these articles, I could not have anticipated such a surge in citations. The statistical article was invited to strengthen the sample size estimation of submitted articles.

Additionally, authors were recruited to write 2 articles on the metaverse to inform readers about recent trends in the educational environment, particularly during the coronavirus disease 2019 pandemic. Citations (159) for these 3 articles accounted for 51.8% of all citations (307). Commissioned articles may not always align with the common interests of readers. However, in recent cases, the editor has been able to focus on topics that interest readers. The editorial office will continue to invite articles addressing topics of widespread concern among readers.

## Is a higher JIF an opportunity for the journal’s promotion or a burden on the editor and management team?

A high JIF value can present an opportunity for JEEHP to become a top-tier journal in the scientific education category. Firstly, it has become eligible for SCIE listing review by the Clarivate editor since its JIF value falls within the 1st quartile of the journal ranking for SCIE journals. Secondly, there may be an increase in submissions from around the world.

Nonetheless, I am concerned about the possibility of receiving too many submissions. On one hand, it is gratifying for the editor to select high-quality manuscripts and publish them on the journal website and other indexing databases such as PubMed, PubMed Central, Scopus, and Web of Science Core Collection, and the quality of manuscripts has improved year by year.

On the other hand, the publishing budget is limited. The editor and editorial management team work on a voluntary basis, and editing is not our primary job. Time is a scarce resource. Moreover, many good or excellent manuscripts might not be considered for peer review due to the limit on the number of publishable articles per year. Apart from the peer-review process and final decision, all other editorial and publishing tasks have been outsourced to commercial publishing companies in Korea, including M2PI (https://m2-pi.com/) for XML, PDF, e-submission system, and journal website management; InfoLumi (https://infolumi.co.kr/) for manuscript editing; Compecs (https://www.compecs.com/) for English proofreading; and Research Factory (https://www.rfactory.kr/) for graphical abstract design. For JEEHP, if the number of articles exceeds 40, covering the costs at a reasonable rate becomes challenging.

## Editor’s bias in selecting manuscripts for peer review

Before any article is published after submission, the editor meticulously reads the manuscript at least 8 times. Another editorial team member also reviews the manuscripts multiple times. The editorial desk screens the submitted manuscripts, and more than 80% are returned without peer review to lessen the burden. This practice saves time for both peer reviewers [3] and editorial team members. Although the editor returns these manuscripts, the reasons are briefly mentioned. The 2 main reasons are misalignment with the journal’s aims and scope and noncompliance with its style and format.

Defining “educational evaluation” is not easy, as it can refer to assessments in various parts of the educational system, including the effectiveness of instructional methods, learning outcomes, curriculum quality, and student performance. Although the aims are broad, the journal’s scope is specified as follows on its website: “adoption of measurement theory to medical health education, promotion of high stakes examination such as national licensing examinations, improvement of nationwide or international programs of education, computer-based testing, computerized adaptive testing, and medical health regulatory bodies.” Some manuscripts have excellent content but do not fit the journal’s style and format. A manuscript that has been returned due to non-adherence to the style and format cannot be considered for peer review again, even if the authors revise and resubmit it.

The minimum requirement for authors is to read the target journal’s instructions for authors or a previous article at least once. JEEHP cannot employ staff to correct manuscripts’ style and format, as there is no full-time staff for journal publishing on the publisher’s side. Some manuscripts are returned despite matching the aims and scope and the style and format due to limited interest from the editor. The editor prefers topics related to recent trends and the journal’s described scope. Whether a manuscript is selected or not depends on the editor’s decision, and some bias may exist in this process.

Additionally, the editor considers manuscripts from middle- or low-income countries more favorably. As a result, at least one manuscript from each country is considered for peer review. Bias from the editor is inevitable in order to save time for editorial team members and reviewers. The journal's scope will be more strictly applied to submitted manuscripts.

## Diamond open access policy by the publisher

The number of diamond open access journals is estimated to be 29,000 in the world [4]. JEEHP is one of them, which means it is a gold open access journal without an author-side article processing charge. The publisher has no intention of changing this policy. To publish more articles through JEEHP, increased budget investment by the publisher is necessary. Until then, the volume of publishing cannot be increased. It is also challenging for the editor and editorial team members to allocate time for the journal, which is why the acceptance rate is so low. Many submitters may be dissatisfied with the “not suitable” comment. The editor apologizes to them for not accepting their high-quality manuscripts.

## Greater emphasis on reporting guidelines for scientific soundness

JEEHP seeks submissions of scientifically rigorous manuscripts that adhere to the following criteria: the research design must be clearly stated in the title and main text [3]; the hypothesis or questions should be explicitly formulated and addressed; adherence to relevant reporting guidelines as outlined by the EQUATOR Network (https://www.equator-network.org/) is required; statistical robustness must be ensured through appropriate sample size estimation [5]; and finally, the interpretation of findings must remain within the bounds of reasonable inference [6]. The editor kindly asks future authors of JEEHP to read the “instructions to authors” and the “aims and scope” more thoroughly; moreover, they should use a template for each reporting guideline when writing a manuscript, available on the journal website. Study designs and corresponding reporting guidelines can be selected following the suggested algorithm [7]. If there is no suitable reporting guideline for the submission, please contact the editorial office at https://jeehp.org/about/contact.php. The editor will consider suggestions for specific reporting guidelines to be included.

The editor hopes that authors’ submissions to JEEHP and their correspondence with the editor will be a pleasant experience for the development of research competence. Additionally, the editor expects that the journal’s excellent JIF, which was announced for the first time, will be helpful for authors in the acknowledgment of their work.

## Figures and Tables

**Table 1.** Highly cited articles of the 2020 and 2021 issues of *Journal of Educational Evaluation for Health Professions* by Web of Science Core Collection journal articles published in 2022 [cited 2023 Jun 28]

Rank	Title	Year	Frequency	Publication type
1	Sample size determination and power analysis using the G*Power software	2021	99	Review
2	Educational applications of metaverse: possibilities and limitations	2021	47	Review
3	E-learning in health professions education during the COVID-19 pandemic: a systematic review	2021	14	Review
4	Effect of virtual reality training to decreases rates of needle stick/sharp injuries in new-coming medical and nursing interns in Taiwan	2020	14	Research article
5	Training in lung cancer surgery through the metaverse, including extended reality, in the smart operating room of Seoul National University Bundang Hospital, Korea.	2021	13	Editorial
6	How to train health personnel to protect themselves from SARS-CoV-2 (novel coronavirus) infection when caring for a patient or suspected case	2020	12	Editorial
7	Malaysian pharmacy students' perspectives on the virtual objective structured clinical examination during the coronavirus disease 2019 pandemic	2021	7	Research article
8	The Effectiveness of cultural competence education in enhancing knowledge Acquisition, performance, attitudes, and student satisfaction among undergraduate health science students: a scoping review	2021	6	Research article
9	Innovative digital tools for new trends in teaching and assessment methods in medical and dental education	2021	5	Educational/faculty development material
10	Husserlian phenomenology in Korean nursing research: analysis, problems, and suggestions.	2020	5	Research article
